# Simultaneous sequencing of genetic and epigenetic bases in DNA

**DOI:** 10.1038/s41587-022-01652-0

**Published:** 2023-02-06

**Authors:** Jens Füllgrabe, Walraj S. Gosal, Páidí Creed, Sidong Liu, Casper K. Lumby, David J. Morley, Tobias W. B. Ost, Albert J. Vilella, Shirong Yu, Helen Bignell, Philippa Burns, Tom Charlesworth, Beiyuan Fu, Howerd Fordham, Nicolas J. Harding, Olga Gandelman, Paula Golder, Christopher Hodson, Mengjie Li, Marjana Lila, Yang Liu, Joanne Mason, Jason Mellad, Jack M. Monahan, Oliver Nentwich, Alexandra Palmer, Michael Steward, Minna Taipale, Audrey Vandomme, Rita Santo San-Bento, Ankita Singhal, Julia Vivian, Natalia Wójtowicz, Nathan Williams, Nicolas J. Walker, Nicola C. H. Wong, Gary N. Yalloway, Joanna D. Holbrook, Shankar Balasubramanian

**Affiliations:** 1 Cambridge Epigenetix Ltd, The Trinity Building, Chesterford Research Park, Cambridge, UK; 2 Cancer Research UK Cambridge Institute, University of Cambridge, Cambridge, UK; 3 Yusuf Hamied Department of Chemistry, University of Cambridge, Cambridge, UK

**Keywords:** Epigenomics, Cancer genomics

## Abstract

DNA comprises molecular information stored in genetic and epigenetic bases, both of which are vital to our understanding of biology. Most DNA sequencing approaches address either genetics or epigenetics and thus capture incomplete information. Methods widely used to detect epigenetic DNA bases fail to capture common C-to-T mutations or distinguish 5-methylcytosine from 5-hydroxymethylcytosine. We present a single base-resolution sequencing methodology that sequences complete genetics and the two most common cytosine modifications in a single workflow. DNA is copied and bases are enzymatically converted. Coupled decoding of bases across the original and copy strand provides a phased digital readout. Methods are demonstrated on human genomic DNA and cell-free DNA from a blood sample of a patient with cancer. The approach is accurate, requires low DNA input and has a simple workflow and analysis pipeline. Simultaneous, phased reading of genetic and epigenetic bases provides a more complete picture of the information stored in genomes and has applications throughout biomedicine.

## Abstract (web summary)

A six-letter sequencing workflow can simultaneously detect genetic and epigenetic bases.

## Main

Information encoded in nucleic acids is fundamental to the biology of living systems. There are multiple dimensions of information stored within DNA. Genetic sequencing of the DNA bases G, C, T and A has been transformed by high-throughput sequencing approaches in the past two decades. Epigenetic information in DNA provides insights into dynamic changes in biology that are closely associated with transcriptional programs^1^ and cell fate^2^. The combination of genetic and epigenetic information provides a more comprehensive view of biology. For instance, the analysis of somatic genetic mutations, together with DNA methylation marks, from blood DNA gave a substantially more accurate prediction of mortality than either genetics or DNA methylation alone^3^. Both DNA methylation and genotype are required to determine the pluripotency of induced stem cells^4^ and their maturation capacity^5^. Germline genetic alterations cause changes in DNA methylation that ultimately dictate predisposition for disease^6–8^. Combining information on DNA methylation with genetic sequence in cell-free DNA (cfDNA) from blood has been shown to substantially increase sensitivity to detect tumor DNA^9^. DNA methylation information can also inform on the tissue of origin of the circulating tumor DNA^10^. Noninvasive prenatal diagnostic analysis has also demonstrated that DNA methylation signal can determine fetal origin of DNA fragments^11^. Epigenetic information in DNA has been retrieved principally via sequencing 5-methylcytosine (5mC). More recently, 5-hydroxymethylcytosine (5hmC) has emerged as an important base modification that can provide information that goes beyond 5mC and genetics^12,13^. Hitherto, researchers have accessed either genetic or epigenetic information, without resolving 5mC from 5hmC.

Commonly used sequencing approaches do not capture full information from both genetics and epigenetics. Next-generation sequencing directly captures the canonical bases G, C, T and A in its readout^14^. A number of base-conversion chemistries have been developed to help differentiate unmodified C from its epigenetic variants, 5mC or 5hmC. These include bisulfite-based approaches such as whole-genome bisulfite sequencing (WGBS)^15^ and bisulfite-free approaches such as enzymatic-methyl sequencing (EM-seq)^16^ and TET-assisted pyridine borane sequencing^17^. An important shortfall of all such methods is that conversion of either the C base, or one of its epigenetic derivatives, to a U (read as T) compromises the direct detection of genetic C-to-T changes, which is the most common mutation in the mammalian genome^18^ and in cancer^19^. Furthermore, the ambiguity caused by C-to-T conversions in the sequenced reads being mapped against either C or T in the reference genome increases false-positive matches in the search space, consequently making computational alignment and mapping of converted reads slower, more expensive and less accurate^20^. Also, these existing methods cannot distinguish 5mC from 5hmC in a single workflow. Methods to distinguish 5hmC from 5mC by exclusively converting only one base have been developed for example oxidative bisulfite sequencing^21^, TET-assisted pyridine borane sequencing-beta^22^, Tet-assisted bisulfite sequencing^23^ and APOBEC-coupled epigenetic sequencing^24^ or by selectively copying 5mC across strands of DNA^25,26^. However, some of these can involve separate, parallel workflows and sequencing to yield full information, which may increase sample requirement, cost and time taken and/or yield data that lack phased information. Combining separate datasets is fraught with difficulties that lead to additive measurement error and coverage gaps across workflows (Supplementary Fig. 1).

In this study, we present a whole-genome sequencing methodology capable of sequencing the four genetic letters in addition to 5mC and 5hmC to provide an accurate six-letter digital readout in a single workflow. The processing of the DNA sample is entirely enzymatic and avoids the DNA degradation and genome coverage biases of bisulfite treatment^27,28^. The method uses all four genetic letters for genomic alignment and encompasses an intrinsic error suppression capability, which leads to high accuracy for both genetic and epigenetic base calling. The approach is versatile and we demonstrate its application to different sample formats, including human genomic DNA and a cfDNA sample from a human patient with cancer.

## Results

**Fig. 1:**
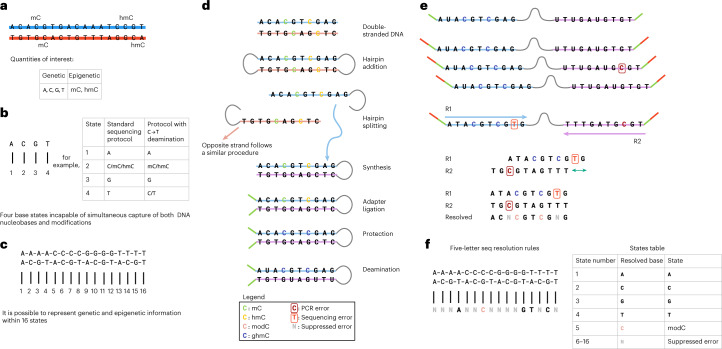
Five-letter seq. **a**, Double-stranded DNA with base modifications. **b**, Traditional genetic sequencing only captures four states of information, which makes it impossible to determine genetic and epigenetic information. Base conversions can alter the information output, but the approach is inherently limited by only having four output states. **c**, Two-base coding results in 4^2^ = 16 possible states enabling simultaneous determination of epigenetic and genetic states. **d**, Laboratory workflow. Hairpins are ligated to double-stranded DNA and the strands are separated. The 5′–3′ strand is omitted for clarity, but follows a similar procedure to the 5′–3′ strand. An additional copy strand is synthesized using Klenow exo-polymerase and short sequencing adapters are ligated. ModCs are protected through oxidation by TET2 and glycosylation by beta-glucosyltransferase (BGT). Treatment by APOBEC3A and UvrD helicase is used to simultaneously open up and deaminate the hairpin. Unprotected Cs are deaminated from C to U (read as T). **e**, Sequencing protocol. The deaminated DNA libraries are PCR amplified and indexes are added. Templates are paired-end sequenced. The two reads represent the same stretch of DNA and are locally aligned. Using a set of resolution rules, the pairs of bases across the two reads are resolved into one of five states: A, C, modC, G, T. The method is able to identify errors occurring during PCR and sequencing. **f**, Overview of the resolution rules and states under the five-letter decoding model. modC is denoted in pink in the diagram and is coded for by the pair CG.

We have developed a method to sequence beyond the four canonical DNA bases and include 5mC and 5hmC (Fig. 1a). The approach is compatible with any sequencer platform. Watson–Crick base pairing provides an explicit digital molecular mechanism for reading up to four letters (or states) (Fig. 1b). To read an epigenetic letter, one can carry out a base-conversion transformation, such as a C-to-T conversion (for example bisulfite seq/EM-seq), where modified Cs are not converted (Fig. 1b). Here, the genetic information is compromised and importantly masks the commonly occurring C-to-T single-base variant. More generally, when sequencing DNA using a single-base coding system with a four-state readout (that is G, C, T, A), one can unambiguously report on a maximum of four states of information in a given run, be those genetic or epigenetic. A system that uses a two-base coding approach, whereby combinations of two bases relay the information for a state, enables up to 16 states to be decoded unambiguously (Fig. 1c). This makes it possible to read all four genetic states and multiple epigenetic states in a single run. We have reduced this to practice for simultaneous five- and six-letter sequencing (seq).

We first describe the five-letter seq workflow that unambiguously resolves the four genetic bases and the epigenetic modifications, 5mC or 5hmC, termed hitherto as modC (unmodified cytosine is referred to as unmodC). In this workflow (Fig. 1d), the sample DNA is first fragmented via sonication and then ligated to short, synthetic DNA hairpin adapters at both ends. The construct is then split to separate the strands. For each original sample strand, a complementary copy strand is synthesized by DNA polymerase extension of the 3′-end to generate a hairpin construct with the original sample DNA strand connected to its complementary strand, lacking epigenetic modifications, via a synthetic hairpin. Sequencing adapters are then ligated to the end. ModCs are enzymatically protected via enzymatic oxidation of 5mC to 5hmC or 5fC or 5caC, by a ten-eleven translocation (TET) methylcytosine dioxygenase 2 mutant^29^ and subsequent enzymatic glycosylation of all 5hmCs, by beta-glycosyltransferase^30^. The unprotected Cs are then deaminated to uracil, which is subsequently read as thymine, via the action of APOBEC3A cytosine deaminase (A3A)^31^ (Fig. 1d). UvrD helicase^32^ is added to the deamination reaction to generate a single-stranded DNA substrate for A3A. The deaminated constructs are no longer fully complementary and have substantially reduced or no duplex stability, and as such can be readily amplified by polymerase chain reaction (PCR). The constructs can be sequenced in paired-end format whereby read 1 (P7 primed) is the original strand and read 2 (P5 primed) is the copy strand. The read data are pairwise aligned so read 1 is aligned to its complementary read 2. Cognate residues from both reads are computationally resolved to produce a single genetic or epigenetic letter (Fig. 1e). Pairings of cognate bases that differ from the permissible five (Fig. 1f) are the result of incomplete fidelity at some stage(s) comprising sample preparation, amplification or erroneous base calling during sequencing. Because these errors occur independently to cognate bases on each strand, most (18 from 24 possible) substitutions result in a nonpermissible pair. Exceptions are changes between A and G on either strand, which could result in genetic (four possible substitutions) or epigenetic (two possible substitutions) miscalls (Supplementary Fig. 2). Nonpermissible pairs are masked (marked as N) within the resolved read and the read itself is retained, leading to minimal information loss and high accuracy at read level. The resolved read is aligned to the reference genome. Genetic variants and methylation counts are produced by read-counting at base level.

The illustrative examples we provide below have used the sequence adapters for the Illumina Novaseq NGS platform; however, other adapters can be readily substituted, and the method is compatible with any sequence reader capable of discriminating at least the four genetic bases.

We performed the five-letter seq workflow on a mixed sample comprising 80 ng of sonicated human genomic DNA from a B lymphoblast cell line (NA12878), obtained from the Genomes in a Bottle project^33^, 0.5 ng bacteriophage λ DNA enzymatically methylated at all cytosines in CpG context by a CpG methyltransferase (M.SssI) and 0.5 ng pUC19 isolated from a methylation-negative strain of *Escherichia coli* (Dam–, Dcm–). DNA was prepared, in duplicate, using the workflow outlined in Fig. 1d and sequenced on an Illumina Novaseq 6000 to produce approximately 550 million paired-end reads. These reads were computationally resolved as outlined in Fig. 1e,f. On average, 98.4% of reads obtained were resolved and of these 89.8% were aligned to the genome with mapq > 0. The average PCR and cluster duplication rate in five-letter seq data was 8.5% with 90.2% of the genome covered by at least one read, and a 15× average coverage of the genome.

Resolved reads contain the four-state genetic information (with the epigenetic information stored as sequence alignment/map format (SAM) tags). Full genetic information enables genomic alignment using standard tools and reduced execution times compared to the three-state alignment necessitated by techniques such as WGBS and EM-seq. The execution time, measured on a single central processing unit (CPU), for genomic alignment of one million resolved single-end 16-state reads using Burrows-Wheeler Aligner-minimal exact match (BWA-MEM) was 7.5 min and the genomic alignment time for one million three-state reads (in 500 K paired-end pairs) using Burrows-Wheeler Aligner-methylation (bwa-meth) was 16.5 min.

We next compared the data quality of both the epigenetic and genetic components of five-letter seq with best-practice methods used to sequence either epigenetics only, or genetics only. To compare the epigenetic component of our method, the same sample mix (80 ng NA12878, 0.5 ng λ and pUC19), was interrogated in duplicate by WGBS (Epitect, Qiagen) and by EM-seq (NEB). For EM-seq and WGBS we used 275 million paired-end reads to generate data at a comparable coverage to the 275 million resolved five-letter seq reads (each paired-end read in five-letter seq yields one single-ended read of equivalent length). Reads were aligned using bwa-meth and methylation was called using MethylDackel, yielding 89.5% and 92% aligned reads (with mapq > 0), and 15× and 17× deduplicated average coverage, for WGBS and EM-seq, respectively. All three technologies had similar coverage and uniformity across the genome. Half-mean coverage was achieved for 87.82% of the bases in the NA12878 genome in five-letter seq, which compares to 85.91% for WGBS and 87.48% EM-seq (Supplementary Fig. 3 and Supplementary Table 1). We observed a small drop in coverage of CpGs near transcription start sites relative to the rest of the genome. Dips and peaks in coverage near transcription start sites have been observed for other technologies^34^. We observed a negligible difference (0.01×) between mean coverage of CpGs in regions with <20% methylation and CpGs in regions with >80% (Supplementary Fig. 4).

**Fig. 2:**
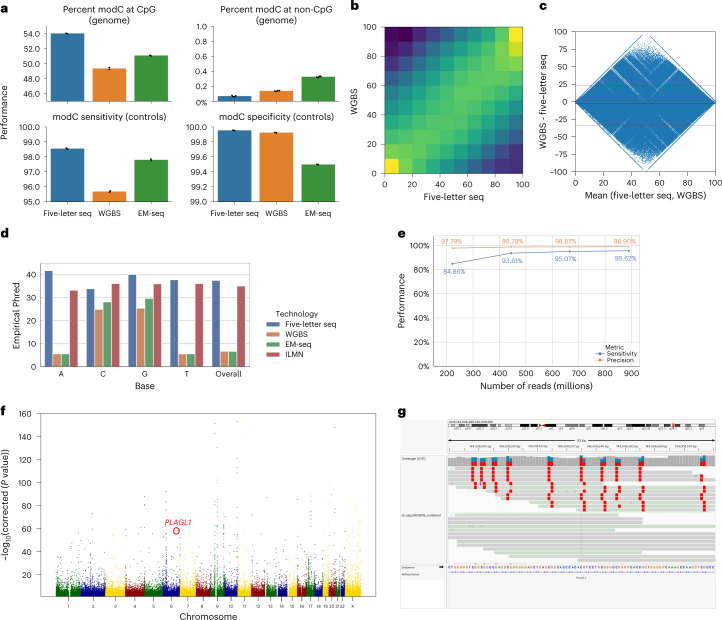
Performance of five-letter seq on genomic DNA. **a**, Top, five-letter seq (blue), WGBS (orange) and EM-seq (green) average modC levels across all autosomes in NA12878 at CpGs in two samples per technology (*n* = 6) (left) and non-CpG contexts—four datapoints correspond to CHH in both samples and CHG in both samples per technology (*n* = 12) (right). Bottom, sensitivity and specificity of modC calling in five-letter seq, as computed on spike-in ground-truth control sequences for both samples (*n* = 6). **b**, Correlation heatmap showing high levels of agreement with WGBS (Pearson’s *R* 0.94, *P* < 10^−8^). Counts were pooled across duplicate samples for both WGBS and five-letter seq and the comparison was limited to sites that were covered at least three times in both methods (26,067,695 sites or 94.24% of all CpGs). **c**, Bland–Altman plot, with the average of the modC levels between the two methods on the *x* axis and the difference on the *y* axis (median difference of −2.6% with 95% of CpGs differing by between −33% and 23%, indicated by solid and dashed red lines, respectively). Counts were pooled across duplicate samples for both WGBS and five-letter seq and the comparison was limited to sites that were covered at least three times in both methods (26,067,695 sites or 94.24% of all CpGs). **d**, Genetic accuracy as calculated on NA12878 high-confidence regions for five-letter seq (blue), WGBS (orange), EM-seq (green) and standard Illumina sequencing (red). **e***,* Precision and sensitivity of variant calling (SNPs and indels) on the *y* axis, using different quantities of five-letter seq reads on the *x* axis, pooled across duplicates. **f**, Manhattan plot of allele-specific methylation in NA12878. The *x* axis is chromosomal location and *y* axis is −log10(p) from Fisher’s exact test of association between genotype and in cis modC levels. *PLAGL1*, a known imprinted gene, is highlighted in red. **g**, Integrative Genomics Viewer (IGV) plot of 92nt region of *PLAGL1* gene centered on a C/T heterozygous SNP. Reads are grouped by the base observed at the variant site and forward and reverse mapping reads are shown in gray and green respectively. ModCs in CpG sites are highlighted in red, with the modification being associated with the G base for reverse reads. Reads exhibiting the (reference) C allele are entirely methylated at CpG sites, whereas reads harboring the T allele are entirely unmethylated.

To compare the accuracy of the genetic sequencing component of our method, the same sample mix was interrogated by Illumina sequencing with standard library preparation using KAPA HyperPrep kit (Illumina) and processed in a similar pipeline. We calculated sensitivity to detect modC (expressed as a percentage) by considering all CpG-context Cs in the lambda genome and evaluating the ratio of modCs to the total number of observed cytosines (modC or unmodC). Similarly, specificity was calculated as the ratio of unmodCs to the total number of cytosines for CpG contexts in the pUC19 reference. Sensitivity of five-letter seq was 98.55% and specificity was 99.95%. This compared well with EM-seq, which had lower sensitivity (97.89%) and lower specificity (99.5%), and to WGBS, which was also less sensitive (95.69%) and less specific (99.92%) (Fig. 2a, bottom). Across the NA12878 human genome, average levels of modC observed at CHG and CHH sites were 0.07% as measured by five-letter seq, 0.14% as measured by WGBS and 0.33% as measured by EM-seq. In contrast, average modC levels at CpG sites was highest as measured by five-letter seq (54.05%), 51.10% as measured by EM-seq and 49.38% as measured by WGBS (Fig. 2a, top). Quantification of modC at all CpGs across reads and at genome level was highly concordant at single-base level between five-letter seq and WGBS (Pearson’s *P* < 0.01, *r* = 0.94) (Fig. 2b). There was a high level of agreement between modC estimates generated from the two methods (Fig. 2c). For the comparisons in Fig. 2b,c, we pooled counts of modified and unmodified C calls at CpGs across both strands in two technical replicates and only considered CpGs, which were covered by at least three reads (94.24% of all CpGs).

To evaluate the genetic component, called bases were compared with the reference at nonvariant sites according to published variant call files for NA12878. Genetic accuracy was calculated as the ratio of correct base calls to total base calls (disregarding N calls). For five-letter seq and Illumina sequencing, the genetic accuracy was consistent across all base types (99.95–99.98%). For WGBS and EM-seq, however, accuracy was high (99.69–99.90%) for C/G bases, but low (around 72.5%) for A/T bases (Fig. 2d). This is caused by unmodC to T deamination in WGBS and EM-seq technologies that results in T bases being called instead of true C bases for the first read in each paired-end read, and in A bases being called instead of true G bases for the second read. This phenomenon has the consequence of masking actual C>T or A>G transitions without additional processing and requires relatively high genomic coverage^35^. A feature of the technology is suppression of PCR and sequencing errors, these suppressed errors constituted 0.45% of bases in the resolved reads (Supplementary Table 1). The accuracy of variant calling was investigated by applying GATK HaplotypeCaller to mapped read files of varying depths (211 million–842 million reads, resulting in mean coverages of 7×–29×). Overall performance (single nucleotide polymorphisms (SNPs) and indels) was determined through comparison with published variant call files (small variants) for NA12878. Sensitivity varied from 84.86% (211 million reads) to 95.62% (842 million reads) with precision consistently above 97.79% (Fig. 2e).

It is important to note that five-letter seq simultaneously determines genetics and epigenetics on the same read. Therefore, combinations of genetic and epigenetic marks in cis on the same DNA molecule are detected. This property can be used to deconvolute genetic and epigenetic properties of heterologous samples. For example, we detected allele-specific methylation in the NA12878 sample. Allele-specific methylation is a wide-spread phenomenon in the human genome whereby DNA methylation is differential between alleles. It can identify regulatory sequence variants that underlie genome-wide association studies signals for common diseases^36,37^. Reads covering a polymorphism were interrogated for methylation levels that differed between alleles. Loci with the strongest effects included human *PLAG1*, a well-known imprinted gene^38^ (Fig. 2f,g).

**Fig. 3:**
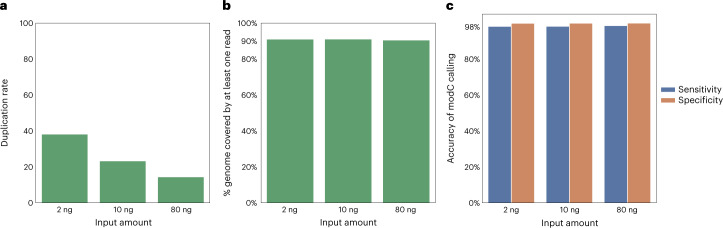
Application of five-letter seq to cfDNA. **a**, Proportion of reads that are PCR and cluster duplicates (y-axis) rates achieved at input of 2 ng or 10 ng of cfDNA or 80 ng gDNA. **b**, Proportion of genome covered with at least one read (y-axis) at input of 2 ng or 10 ng of cfDNA or 80 ng gDNA. **c**, Sensitivity and specificity of modC detection is unaffected by input amount. Input of 0.5 ng spike-in ground-truth control DNAs for the gDNA samples and 0.05 ng for the cfDNA samples, sensitivity on methylated lambda DNA in blue and specificity on unmethylated pUC19 DNA in orange.

The analysis of cfDNA is a burgeoning aspect of diagnostics and application areas include enhanced noninvasive prenatal diagnosis, early cancer detection and disease monitoring^39^. A practical challenge is to work with the limiting amount of cfDNA that can be extracted from a standard blood draw, which is typically around 10 ng/ml (ref. ^40^). An accurate sequencing method that can simultaneously detect genetic and epigenetic information from the same sample could transform cfDNA analysis. We deployed the five-letter seq workflow (Fig. 1d,e) for the analysis of cfDNA from an individual with stage III colon cancer. Input DNA quantity was 2 or 10 ng of cfDNA or 80 ng of genomic DNA (gDNA), in each case mixing the unsonicated sample with λ and pUC19 DNA spike-in controls, as previously described. PCR and cluster duplication rates ranged from 38.2% to 14.5% as input DNA increased from 2 to 80 ng (Fig. 3a), with 90.85%, 90.89% and 90.35% of the genome covered with at least one read, at mean read depth of 21.8×, 27.6× and 30.4×, for 2, 10 and 80 ng input, respectively (Fig. 3b and Supplementary Table 2). The accuracy of methylation detection, determined for the λ and pUC19 DNA controls, remained high achieving above 98% sensitivity and specificity 99.89%, 99.94% and 99.98% (Fig. 3c), even at 0.05 ng of control DNA in the 2 ng mixed sample. The fragment length distribution typical of cfDNA is retained in the five-letter seq library suggestive of profiling of the mono and dinucleosomal fractions (Supplementary Fig. 5).

**Fig. 4:**
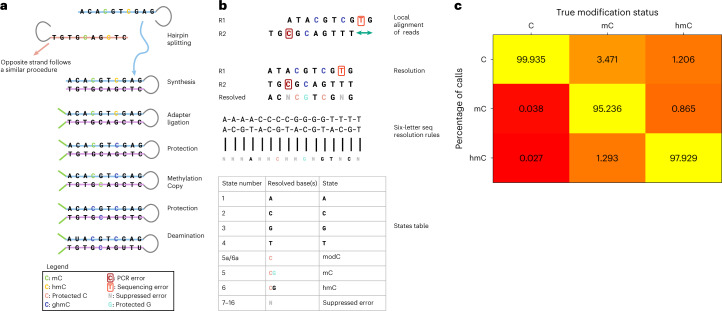
Six-letter seq. **a**, Schematic of six-letter epigenetic sequencing protocol. A similar protocol to that of five-letter seq, described in Fig. 1d, is followed with the addition of a methyl-copy step where DNMT5 copies the 5mC from the original to the copy strand. The 5hmC is protected by glycosylation and not copied. **b**, Overview of the resolution rules and states under the six-letter decoding model. A protected C on the original strand, signifying modC, is denoted by pink in the diagram and table; a G opposite a protected C on the copy strand is denoted by light blue. The 5mC is denoted by a protected (pink) C followed a protected (blue) G and 5hmC is denoted by a protected (pink) C followed by an unprotected (black) G. **c**, Call-rate matrix, which contains the rate at which six-letter seq calls unmodC, 5mC and 5hmC when the true state is unmodC, 5mC and 5hmC. This is estimated from ground-truth control sequences for which the modification status of each CpG is known. We calculate the rate at which six-letter seq calls unmodC, 5mC and 5hmC at unmodCpGs on a fully unmethylated pUC19 (first column), the rate at which six-letter seq calls unmodC, 5mC and 5hmC at 5mCpGs on a fully methylated lambda genome (second column), and the rate at which six-letter seq calls unmodC, 5mC and 5hmC at 5hmCpGs on a synthetic oligonucleotide (third column).

The enzymatic oxidation of 5mC generates 5hmC^41^, which has been shown to have value as a marker of biological states and disease, including early cancer detection from cfDNA^42,43^. We have adapted our platform to enable six-letter seq of DNA that comprises G, C, T, A, mC and hmC (Fig. 4a). A critical requirement is to disambiguate 5mC from 5hmC without compromising genetic base calling within the same sample fragment. The first three steps of the workflow are identical to five-letter seq shown in Fig. 1d to generate the adapter ligated sample fragment with the synthetic copy strand. To enable six-letter seq, critically, methylation at 5mC is enzymatically copied across the CpG unit to the C on the copy strand using DNA methyltransferase 5 (DNMT5), whereas 5hmC is glycosylated via beta-glycosyltransferase to prevent such a copy. DNMT5 has been selected to perform the copy step because of its specificity for copy methylation over de novo methylation^44^. The unmodCs are then deaminated to uracil, which is subsequently read as thymine via the joint action of TET2, A3A and UvrD helicase. Glycosylation still protects 5hmC. The DNA is subjected to PCR amplification and sequencing as described earlier (Fig. 1e). The reads are pairwise aligned and resolved using the two-base code shown in Fig. 4b. Each of unmodC, 5mC and 5hmC can be resolved as the three CpG units have distinct sequencing readouts of the two-base code (Fig. 4b). In this workflow modC can also be detected in non-CpG contexts, although in such contexts 5mC cannot be distinguished from 5hmC. The evidence suggests this is not such a substantial limitation as conversion of 5mC to 5hmC by TET, has been shown to be minimal outside of CpG context^45^ and so it would be a reasonable assumption that the majority of modC calls at CHH sites are 5mC.

We estimated the accuracy of base-modification status calls produced by six-letter seq using ground-truth control sequences with known modifications. We used the same fully unmethylated pUC19 and fully methylated lambda as were used in the evaluation of the five-letter seq protocol and an additional short synthetic oligonucleotide with 5hmC present at specific CpGs. Six-letter seq correctly identified 95.24% 5mC and 97.93% 5hmC status calls, across all reads aligning to those CpGs (Fig. 4c) and retained a very high (99.93%) accuracy of detecting unmodC across all reads. Extrapolation of this accuracy to genome-scale is detailed in Supplementary Fig. 6.

## Discussion

We have described a sequencing platform that will deliver the full complement of genetics in addition to the epigenetic bases 5mC and 5hmC at base resolution in a single workflow and data pipeline. The platform operates via a two-base coding mechanism at the molecular level coupled with decoding software. It also improves the accuracy of genetic sequencing and variant calling alongside high-accuracy epigenetic base calling. The platform comprises an all-enzyme workflow with extremely high conversion efficiencies, thus enabling accurate data from valuable, low-input biological samples or from clinical cfDNA. The readout of genetic or epigenetic bases is digital and context independent. The alignment uses a four-base system that is faster and more accurate than three-base alignments used for bisulfite sequencing or EM-seq. The acquisition of accurate, phased genetic and epigenetic information, in a single experimental and data workflow is likely to offer numerous advantages in research and in diagnostics by providing more comprehensive biological information, with ease of use and at reduced cost.

Third-generation sequencing systems^46,47^ also measure epigenetic and genetic modifications in the same workflow by detecting signal differences directly caused by epigenetic modifications. As such, they are incompatible with PCR amplification of the starting material, as DNA modifications such as methyl and hydroxymethylcytosine are lost during PCR. Therefore, they are severely limited by the read depth achievable at low DNA inputs. For instance, Katsman et al.^48^ described nanopore sequencing of 15–60 ng of isolated cfDNA, achieving an average sequence depth of 0.2×. This low sequence depth precludes any sampling of the circulating tumor DNA (which is a small fraction of cfDNA) and quantitation of epigenetic modifications at specific loci beyond 0% or 100% methylated. It also requires reliance on read-level genetic accuracy without consensus across multiple reads, this is estimated on both the promethION and minION as <99%^49^.

The method presented in this study, due to its use of base conversion, is compatible with PCR. In Supplementary Table 2 we show 22× sequencing depth achieved from 2 ng of cfDNA with only 38% duplication rate, further depth could be achieved before the library is saturated. The 22× enables sampling of cfDNA (including genetic states and epigenetic states) present as <5% of total library and quantification of epigenetic state (resolving differences of <5%). Read-level genetic accuracy in this technology is high (99.97%) due to the inherent error suppression technology, and the ability to produce high depth at low input, allows consensus variant calling at least equal to Illumina accuracy (Fig. 2e).

## Conclusions

The most prevalent epigenetic modifications to DNA in mammalian biology are 5mC and 5hmC. There are other, less frequent, modifications such as 5-formylcytosine, 5-carboxycytosine and N^6^-methyladenine, in various organisms. The measurement of additional modifications by exploiting additional information states inherent in the technology will be the subject of future work, beyond the scope of this manuscript. As the platform fundamentally uses Watson–Crick base pairing to decode information, it can be made readily compatible with any sequencer platform and we see opportunities for its future application to long-read sequencing and single-cell analysis.

## Methods

### Samples

Genomic DNA was obtained from the Coriell Institute (https://www.coriell.org/0/Sections/Search/Sample_Detail.aspx?Ref=NA12878).

Double spun plasma was obtained from Geneticist Inc. from a single woman patient with a gastric cancer diagnosis. cfDNA was extracted using the NextPrep-Ma kit on the Chemagic Prime platform (Perkin Elmer chemagen Technologie GmbH).

### Ground truth controls

An 80-base pair (bp) oligonucleotide with balanced CG content and no homology to human or lambda genome or pUC19, and symmetrical 5hmC at one CpG and asymmetrical 5hmC at another, was designed and purchased from ATDBio Ltd. (Supplementary Fig. 7). The oligonucleotide pair was diluted in 100 mM potassium acetate, 30 mM HEPES, pH 7.5 (IDT) and annealed by heating to 94 °C for two minutes and cooling gradually to 4 °C.

For unmethylated pUC19 control, the plasmid (NEB) was transformed into *dcm-/dam- E. coli* chemically competent cells (NEB). Plasmid was isolated from culture using a QIAprep Spin Miniprep Kit (Qiagen). Methylated lambda DNA was prepared using unmethylated lambda DNA (Promega). Briefly, 1 μg DNA was incubated with eight units of CpG methyltransferase MSssI (NEB), NEB buffer 2, 320 μM S-adenosyl-methionine (NEB) at 37 °C. After four hours, a further two units of MSssI was added and the reaction incubated for a further four hours at 37 °C. The DNA was purified using a Zymo clean and concentrator column. Complete methylation was checked using methylation sensitive HpaII (NEB) and methylation insensitive MspI (NEB). A negative control using unmethylated lambda DNA was also prepared and used to check digestion with MspI and HpaII. Both methylated lambda DNA and unmethylated pUC19 were diluted in 10 mM Tris-HCl pH 8.0, 0.1 mM EDTA and fragmented to ~250 bp using a Covaris M220.

### Laboratory processing

For five- and six-letter seq, variable amounts of input material were used according to the manufacturer’s protocol (Cambridge Epigenetix). Genomic DNA was sonicated using the Covaris M220 Sonicator set to a target size of 250 bp. Other target sizes are compatible with the method and achieve similar yields (Supplementary Table 3). The fragment length profile of cfDNA is conserved in the sequencing libraries (Supplementary Fig. 5).

For five-letter seq, cfDNA or sheared gDNA was mixed with 1 µl of spike-in control (at 0.5 ng/μl for main DNA inputs of ≥80 ng and above and 0.05 ng/μl for inputs ≤10 ng), 3.5 µl End Prep reaction buffer and 1.5 µl End Prep Enzyme Mix for end repair and A-tailing (catalog no. E7647, NEB). The reaction was incubated at 20 °C for 30 min followed by 65 °C for 30 min. In the same mix, Adapter 1 (ATGACGATGCGTTCGAGCATCGUCAUT, all Cs are methylated, Biomers.net GmbH) was ligated using 3.75 µl of adapter 1, 0.5 µl of ligation enhancer and 15 µl of ligation master mix (both catalog no. E7647, NEB) and incubated at 20 °C for 15 min. Afterwards SPRIselect magnetic beads (catalog no. B23319, Beckman Coulter) were added to the solution and a clean-up performed according to the manufacturer’s protocol. The library was eluted in 23.75 µl nuclease-free water (catalog no. W4502, Sigma) and 3 µl of 10× rCutsmart buffer (catalog no. B6004S, NEB) and 3.25 µl USER (catalog no. M5505, NEB) were added. This was incubated for 30 min at 37 °C. Next the DNA was purified using SPRI magnetic beads (catalog no. B23319, Beckman Coulter) using the manufacturer’s protocol. DNA was eluted in 12 µl Library Elution buffer (10 mM Tris-HCl pH 8.0) and used subsequently in strand synthesis. Here, 2 µl of each of 10× NEB buffer 4 (catalog no. B7004S, NEB), dNTPs (10 mM each, catalog no. R0192, Thermo Fisher Scientific), Klenow (exo-) (catalog no. P7010-LC, Thermo Fisher Scientific) and T4 PNK (catalog no. EK0032, Thermo Fisher Scientific) were added and incubated for 30 min at 37 °C. The reaction was then exposed to denaturing at 95 °C for two minutes and gradually cooled (at −0.1 ºC/second) to enable reannealing. Immediately afterwards, 2.5 µl adapter 2 was ligated (forward: ACACTCTTTCCCTACACGACGCTCTTCCGATC*T, *indicates phosphorothioate, reverse: GATCGGAAGAGCACACGTCTGAACTCCAGTCA, all Cs are mC, Biomers.net GmbH), 0.5 µl of ligation enhancer, 15 µl of ligation master mix (both catalog no. E7647, NEB) and 12 µl nuclease-free water (catalog no. W4502, Sigma). This was incubated for 15 min at 20 °C. Next, the DNA was purified by another SPRIselect bead purification. Elution from beads was performed in 30 µl and then 10 µl of reconstituted TET2 5× supplement buffer (10 mM a-Ketoglutarate, 0.25 M Tris-HCl pH 8.0, 10 mM ATP), 1 µl of UDP glucose, 1 µl of T4 beta-glucosyltransferase (both catalog no. EO0831, Thermo Fisher Scientific), 1 µl of 100 mM DTT and 2 µl of TET2 (Cambridge Epigenetix) were added. After mixing 500 mM Fe(II) sulfate hexahydrate (Sigma) 1:1250 with nuclease-free water (catalog no. W4502, Sigma), 5 µl of this dilution was added to the DNA and incubated for 60 min at 37 °C. Next, the converted DNA was purified by a SPRIselect bead clean-up according to the manufacturer’s protocol, eluted in 31 µl of nuclease-free water and exposed to another enzymatic conversation reaction. For this, 12.75 µl nuclease-free water, 17.5 µl 4X APOBEC buffer (200 mM BisTris pH6.1, 0.4% Tween), 1.75 µl 100 mM ATP (catalog no. A6559, Sigma), 3.5 µl 100 mM MgCl2 (catalog no. M1028, Sigma), 2 µl APOBEC-A3A (Cambridge Epigenetix) and 2.5 µl of UvrD Helicase (Cambridge Epigenetix) were added. The reaction was incubated for 1.5 h at 37 °C and cleaned up afterwards with another SPRIselect bead-mediated purification according to the manufacturer’s protocol. The DNA was eluted in 20 µl nuclease-free water and amplified by PCR using 5 µl of Abclonal Unique Dual Index Primers for Illumina (Abclonal no. RK21624_SetA) and 25 µl of 2X KAPA HiFi U^+^ Polymerase (no. KK2802). For 80 ng of gDNA, six cycles of PCR were used, whereas 10 ng of cfDNA used seven cycles of PCR and 2 ng cfDNA used eight cycles (PCR program: 30 seconds at 98 °C for initial denaturation, 10 seconds at 98 °C for denaturation, 30 seconds at 62 °C for annealing, 60 seconds at 65 °C for extension and five minutes at 65 °C for final extension. After PCR the final libraries were purified by SPRIselect bead, eluted in 15 µl Library Elution buffer (10 mM Tris-HCl pH 8.0) and quantified using TapeStation D5000 reagents (Agilent).

For six-letter seq, the five-letter seq protocol has been used with the following modifications. For the PNK/Klenow step, two additional components are added to the enzyme mix, 2.5 µl UDP glucose and 1 µl T4 beta-glucosyltransferase (both catalog no. EO0831, Thermo Fisher Scientific). After adapter 2 ligation, an additional protocol step is performed. In this additional step, 15 µl of DNA is combined with 3.95 µl of nuclease-free water (catalog no. W4502 Sigma), 0.75 µl of 100 mM ATP (catalog no. A6559, Sigma), 1.5 µl of 100 mM MgCl_2_ (catalog no. M1028, Sigma), 0.4 µl of S-adenosylmethionin (catalog no. B9003S, NEB), 6 µl of 5X DNMT buffer (250 mM Tris-HCl pH 8.0, 10 mM DTT) and 2.4 µl of DNMT5 (Cambridge Epigenetix). This was incubated for 3 h at 37 °C.

To generate standard genomic libraries the KAPA Hyper Prep kit (catalog no. KK8500, Roche) was used according to the manufacturer’s protocol with the following modifications. Using a Covaris M220 sonicator, 100 ng of genomic DNA (Coriell, catalog no. NA12878) was sonicated to ~250 bp in 50 µl sonication tubes (Covaris) and used in the experiment. For adapter ligation, standard TRUSeq adapters were used (15 µM, Illumina). Ampure beads (Beckman Coulter) were used instead of KAPA clean-up beads and the elution volume was reduced to 21 µl using library dilution buffer (10 mM Tris-HCl, pH 8.0). For library amplification the KAPA Hifi Hot start PCR kit was used (catalog no. KK2500, Roche) according to the manufacturer’s protocol using four PCR cycles and 1 µM final primer concentration. Final libraries were quantified using D1000 HS screen tapes (Agilent).

EM-seq libraries were produced using the New England Biolabs EM-Seq kit (catalog no. E7120, NEB) according to the manufacturer’s protocol with the following modifications. Instead of using the EM-seq control DNA, the ground-truth spike-in DNAs described in above were used to allow a direct comparison. Denaturation was performed using Formamide (catalog no. F9037, Sigma). For the final PCR, seven cycles of PCR were used (80 ng gDNA input). Libraries were quantified using D1000 HS screen tapes (Agilent) on a Tapestation (catalog no. 4200, Agilent).

Before WGBS, the libraries were prepared using the EM-seq kit (catalog no. E7120, NEB) and the EpiTect Plus DNA Bisulfite kit (catalog no. 59124, Qiagen) with the following modifications. Instead of using the EM-seq control DNA, the ground-truth spike-in DNAs described above were used to allow a direct comparison. The EM-seq protocol was followed until Clean-Up of Adapter Ligated DNA and the eluate was then used as input for the EpiTect Plus DNA Bisulfite kit. The high-concentration sample setup was used for bisulfite conversion. To improve bisulfite conversion, the 20 µl elution was used for another round of conversion using the EpiTect Plus DNA Bisulfite kit (catalog no. 59124) again. After the second round of conversion, the DNA was PCR amplified using the EM-seq kit according to the manufacturer’s protocol starting from PCR amplification using eight cycles.

Sequencing was performed using the S4 Standard workflow on the Illumina NovaSeq. Libraries were quantified using the Tapestation D5000 (Agilent) and subsequently diluted to 1.2–1.8 nM for loading on the sequencer. To balance out the low cytosine content in deaminated libraries (WGBS, EM-seq, five-letter seq and six-letter seq), 8% PhiX (catalog no. FC-110-3001, Illumina) was added according to the manufacturer’s protocol. All libraries were run in a paired-end set up using either 200 or 300 cycle kits in a 111/8/8/111 or 151/8/8/151 base-reads setup.

### Downsampling

To obtain data that were comparable across the different technologies, five-letter seq data was downsampled to 550 million paired-end reads and EM-seq and WGBS data was downsampled to 275 million paired-end reads. Downsampling was achieved using seqtk (https://github.com/lh3/seqtk).

Additionally, five-letter seq data were trimmed using flexbar (https://github.com/seqan/flexbar) from PE151 to PE111 to match the read length of the EM-seq and WGBS data.

### Data processing of five- and six-letter seq

FASTQ files were trimmed and quality-filtered using fastp^50^ before being processed through a resolution algorithm designed by Cambridge Epigenetix. The resolution algorithm corrects for any misalignment of the original and copy strands using a modified Needleman–Wunsch pairwise alignment. Errors identified by unexpected pairings of bases between the original and copy strand are suppressed in the resolved FASTQ file by being converted to N. Phred scores for resolved bases are determined using empirically calculated tables of quality scores that are both instrument and read-length specific. Read-pairs that failed to resolve, defined as having more than 5% unexpected base pairing, indicating they did not derive from the expected hairpin-connected original and copy strand constructs, were filtered out. The resolution approaches for five- and six-letter seq libraries differ merely by the resolution rules within this algorithm; resolution rules are described in Figs. 2 and 4.

Resolved FASTQ files were aligned using BWA-MEM^51^ to a standard four-letter reference genome comprising of both GRCh38 and spiked-in control sequences. Epigenetic information encoded in tags in the resolved FASTQ files was passed on into the aligned BAM files and stored using the MM tag. The aligned BAM files were then split into reads aligning to the genome and reads aligning to the controls; unmapped reads were filtered out. Reads aligning to the genome, to the methylated bacteriophage lambda control and to the unmethylated pUC19 control were deduplicated using Picard MarkDuplicates^52^. Reads aligned to the controls were downsampled to a mean coverage of 200× on each control genome before being deduplicated. A range of standard metrics were calculated on the genome-aligned reads using samtools^53^, Qualimap^54^, deepTools^55^ and Picard^56^. Accuracy of the genetic base calling was calculated relative to the known genotype of high-confidence regions of chromosome 20 of the NA12878 sample. Quantification of epigenetic modifications was calculated at each CpG, CHG and CHH site that was present in the reference genome and covered in the sequencing. This was performed using software developed by Cambridge Epigenetix. Likewise, quantification of epigenetic modifications was calculated at each CpG site in the methylated bacteriophage lambda control and the unmethylated pUC19 control. Sensitivity of modification calling was calculated from the methylated bacteriophage lambda control and specificity of modification calling was calculated from the unmethylated pUC19 control.

All of the processing was performed using a software pipeline developed by Cambridge Epigenetix, written in the Nextflow orchestration language and processed on the Google Cloud Platform (GCP).

### Data processing of EM-seq and WGBS samples

Processing of EM-seq and WGBS samples was performed using a software pipeline written in the Nextflow orchestration language and processed on the GCP. Trimming of FASTQ files was performed using Trim Galore!^56^. Alignment to a deaminated reference genome comprising of both GRCh38 and spiked-in control sequences was performed using bwa-meth^57^ and deduplication was performed using Picard MarkDuplicates. Modification calling at CpG, CHG and CHH sites was performed using MethylDackel^58^.

### Data processing of Illumina sequencing samples

Processing of Illumina samples was performed using a software pipeline written in the Nextflow orchestration language and processed on the GCP. Trimming was performed using fastp. Alignment to a GRCh38 reference genome was performed using BWA-MEM and deduplication was performed using Picard MarkDuplicates.

Alignment runtimes were compared by first subsampling five-letter seq samples to one million reads and subsampling EM-seq, WGBS and Illumina sequencing reads to 500,000 reads. Alignment runtimes were then calculated by timing an alignment running on a single central processing unit (CPU).

### Genetic accuracy metrics

Genetic accuracy metrics were computed using a table of empirical Phred scores. An empirical Phred score is a measure of genetic accuracy, expressed as a Q-score, evaluated by comparing observed read data to a truth set. In the table of Phred scores, empirically computed Phred scores and numbers of raw counts of correct and incorrect observations are stratified by base and nominal, that is instrument reported, Phred score. Empirical Phred scores were computed for five-letter seq, WGBS, EM-seq and Illumina sequencing, by considering NA12878 sequence data from chr20 for each of the technologies. The truth set was derived by masking the hg38 reference genome fasta file by the gold-standard Genome in a Bottle variant call data^58^ using the Bedtools maskfasta command (v.2.25.0). Additionally, within chr20, only high-confidence regions, as defined by Genome in a Bottle, were considered. To compute empirical Phred scores, a Python script was used to query the read data in a pileup-oriented fashion using pysam (v.0.19.1). For each pileup, a reference base was defined (unless masked) and correct (matching) and incorrect (nonmatching) observations were tallied. N bases, both in the reference and in the observed read data, were ignored. For completeness, the observations were stratified by observed base and nominal Phred. Finally, Phred scores were computed using the equation −10 × log10(1−(no. correct/(no. correct + no. incorrect))) and rounded down to the nearest integer. In the case where no incorrect observations were made, a maximal Phred score of 60 was assigned.

Genetic accuracies were computed for each of the four technologies by considering the base and nominal Phred stratified correct and incorrect base-call counts from the empirical Phred score table. To this end, genetic accuracies were computed for each base type (A, C, G, T) by considering only count data from bases with a nominal Phred score greater than or equal to 25. This threshold allowed for evaluating genetic accuracies while avoiding data that either of the technologies classified as poor. Genetic accuracy was defined by the equation no. correct/(no.correct + no. incorrect). Overall genetic accuracies were computed by tallying counts across base types.

To examine variant calling performance, the five-letter seq reads from two 80 ng gDNA (NA12878) samples were pooled using samtools merge (v.1.15.1). The combined.bam file had a total read count of 842 million reads, equivalent to a mean coverage of 28.6×. The combined.bam file was then downsampled into four separate.bam files representing fractions of 0.25, 0.50, 0.75 and 1.0 times the original depth. This was achieved using samtools view–subsample {fraction}–subsample-seed 1 and resulted in.bam files of 211 million reads (7.1×), 421 million reads (14.3×), 632 million reads (21.5×) and 842 million reads (28.6×) respectively. Variant calling was then performed for each of these samples by GATK HaplotypeCaller (v.4.2.5.0) with default settings. For efficiency, HaplotypeCaller was run in parallel for each chromosome and the resulting VCF files were merged using GATK MergeVCFs. Finally, RTG Tools’ vcfeval function (v.3.12.1) was used to compare the five-letter seq variant call data to a ground-truth set defined by the Genome in a Bottle VCF file for NA12878 (https://ftp-trace.ncbi.nlm.nih.gov/giab/ftp/release/NA12878_HG001/NISTv3.3.2/GRCh38/). Evaluation was constrained to the high-confidence regions defined by the associated NA12878.bed file (same location). Overall variant calling performance, representing performance across both SNPs and indels, was extracted from the summary.txt output file. Reported sensitivity and precision metrics were associated with receiver operator curve score thresholds resulting in maximal F-measure.

### Call-rate matrix for six-letter seq

A call-rate matrix is used to measure the accuracy of modC calls. Each cell in the matrix *M* represents the rate at which the method calls a particular modification X when the true modification is Y, for example, the cell *M(unmodC, mC)* represents the rate at which a method calls unmodC when the true modification status is modC. Each column of the matrix, corresponding to the rate at which we call each modification status for a particular true modification status, is estimated using a different spike-in control: a fully unmethylated pUC19 for the column corresponding to a true state of unmodC, a fully methylated lambda for the column corresponding to a true state of 5mC and a synthetic oligonucleotide for the column corresponding to a true state of 5hmC. For a given column, the rate is calculated as the proportion of bases with each modification status in the set of bases for which the genetic base call is C and which are aligned to CpGs in the given control sequence.

### Allele-specific methylation calling

SNP calling was done using GATK (v.4.2.6) HaplotypeCaller. The resulting VCF file and the BAM file generated from the five-letter seq pipeline described above were parsed using pysam (v.0.19.1). For each heterozygous SNP site, a script counted the number of times a modC or unmodC call was associated with a CpG in a read containing each allele. Reads that overlapped with the variant site but did not contain a base aligned with the variant site or for which the base call did not match either of the two alleles were not included in these counts. Only sites that had at least six reads containing each allele were considered for ASM calling. The significance of the association was determined using Fisher’s exact test based on a contingency table of counts (variant alleles on one axis and unmodC/modC on the other). After calculating *P* values for all heterozygous variant sites that had sufficient reads for each allele, Benjamini–Hochberg’s correction was applied to minimize the false discovery rate; this was calculated using statsmodels (v.0.13.2).

### Reporting summary

Further information on research design is available in the Nature Portfolio Reporting Summary linked to this article.

## Online content

Any methods, additional references, Nature Portfolio reporting summaries, source data, extended data, supplementary information, acknowledgements, peer review information; details of author contributions and competing interests; and statements of data and code availability are available at 10.1038/s41587-022-01652-0.

### Supplementary information

Supplementary Information Supplementary Figs. 1–7 and Tables 1–3.

Reporting Summary

## Data Availability

Data described in this study are available from National Center for Biotechnology Information National Library of Medicine, Gene Expression Omnibus with no restrictions or conditions on access GSE208549 (ref. ^59^).

## Code availability

Source code for procedures described in this study is available in a public repository on github with no restrictions or conditions on access, https://github.com/cegx-ds/couplet.
