# Supplementary material for: Simultaneous sequencing of genetic and epigenetic bases in DNA
Source: Nat Biotechnol. 2023 Feb 6;41(10):1457–64. doi: 10.1038/s41587-022-01652-0 (PMC10567558; doi:10.1038/s41587-022-01652-0)
Supplement: Supplementary file 1 — Supplementary Figs. 1–7 and Tables 1–3. [file 41587_2022_1652_MOESM1_ESM.pdf]

nature biotechnology

Article https://doi.org/10.1038/s41587-022-01652-0

Simultaneous sequencing of genetic and epigenetic bases in DNA

In the format provided by the authors and unedited

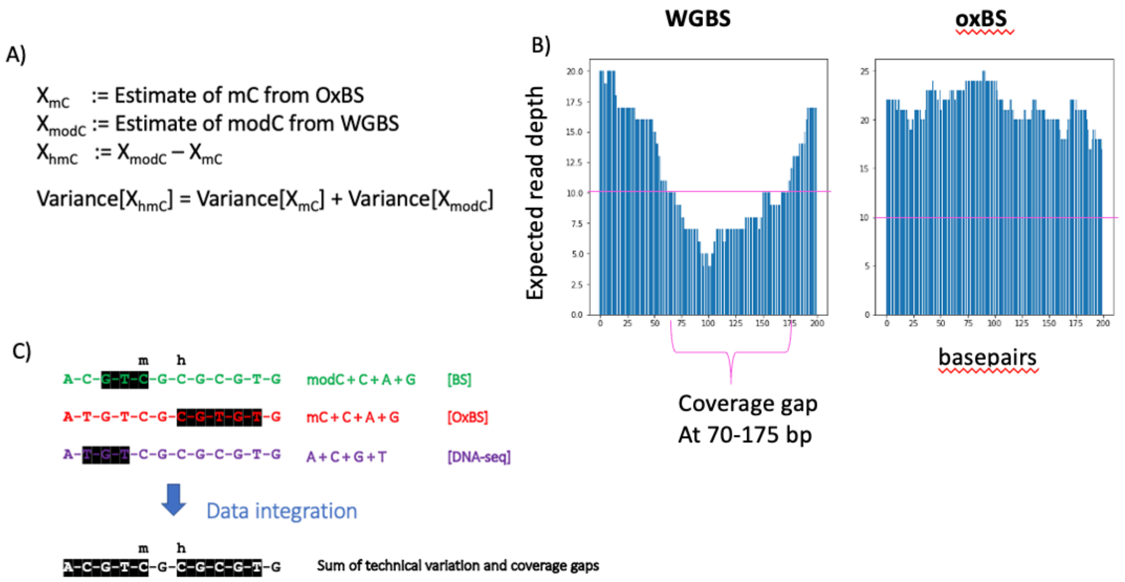

Supplementary Figure 1 To obtain genetics, mC and hmC using existing methods, it is necessary to combine information from a standard whole genome sequencing experiment with data from separate WGBS and OxBS experiments. This presents a number of practical challenges. **SFig 1A)** illustrates how the variance in estimates for hmC levels at a given CpG is the sum of the variance of our estimates of modC from WGBS and mC from OxBS. **SFig1B)** contains an example of expected coverage (reads sampled uniformly from a genome) at the same 200nt region across two simulated sequencing experiments with average 20x coverage; the left is a WGBS experiment and the right is an oxBS experiment. Whenever the coverage dips below 10x for either of the two – no call on mC or hmC can be made. **SFig1C)** provides a schematic illustrating how areas of low coverage can combine unfavourably when combining data across multiple experiments. The integrated dataset will have a coverage gap (denoted by black shading) in any region which has insufficient coverage in any one of the experiments being combined.

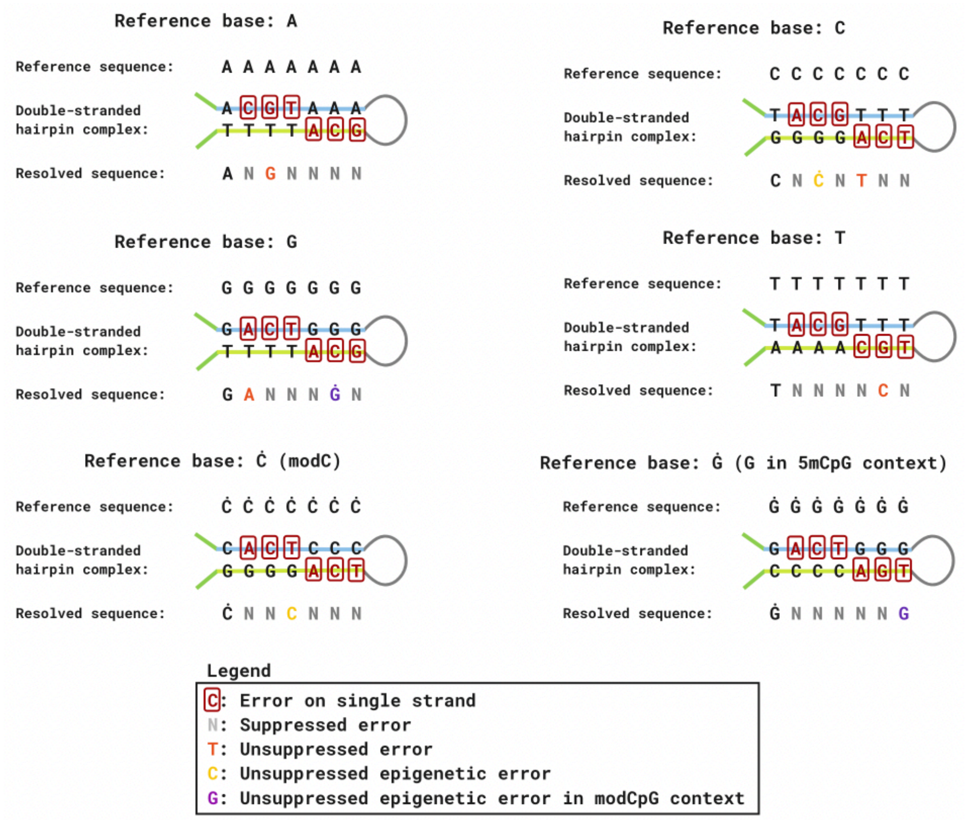

Supplementary Figure 2 Error suppression in 6-Letter sequencing. The sequencing technology employs a two-base coding approach for resolving DNA bases. As an example, a resolved A is coded by an A on read 1 and a T on read 2. PCR and sequencing errors occur independently on the original and copy strands, changing the two-base code. Of the sixteen possible two-base combinations, six code for epigenetically modified or unmodified bases and ten are the result of errors occurring on either strand, these being suppressed to an N state during resolution. The figure outlines all possible single-strand base errors and their impact on the resolved sequence. Each subpanel represents a reference base: A, C, G, T, Ċ (modC) and Ġ (denoting a G found only in 5mCpG context). The first two-base combination is permissible, i.e. it is the two-base code that resolves to the reference base. The following three combinations represent errors to the base on the original strand (read 1). The final three combinations represent errors to the base on the copy strand (read 2). When the resolved base is an N, the error has been suppressed. Of the 36 possible error-driven alterations from the six permissible two-base combinations, caused by an error on either strand, 32 are suppressed in a genetic context. The four error-driven alterations (shown in orange) that result in a genetic miscall are caused by an A<-->G error in read 1 or read 2. There are four sources of epigenetic miscalls (yellow and purple) of which two (purple) only impact epigenetic calls in modCpG context (as G and Ġ are responsible for differentiating 5mC and 5hmC). In 5-Letter sequencing mode, the errors to and from the Ġ base (shown in purple), are not relevant resulting in only two sources of epigenetic errors (shown in yellow).

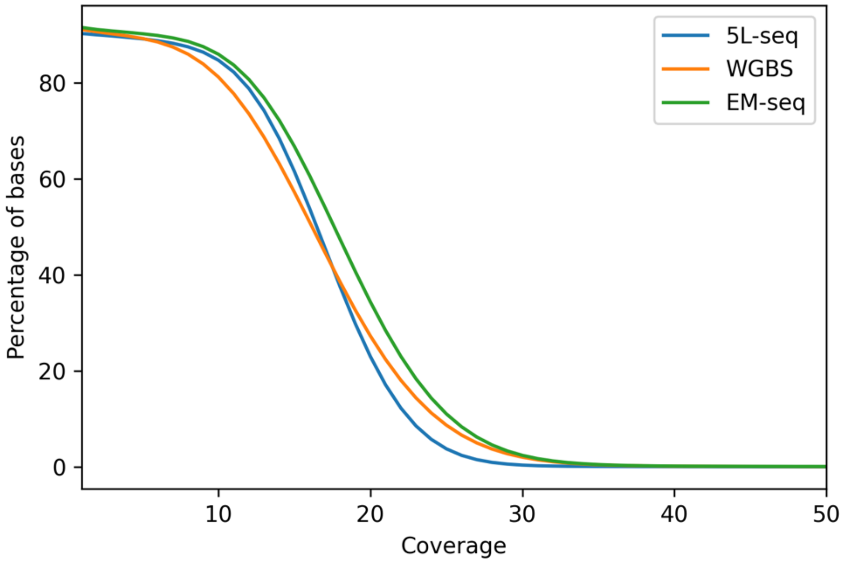

Supplementary Figure 3

Proportion of bases in the NA12878 genome achieving coverage by read depth is plotted for the 3 methodologies, computed using the bamqc tool in Qualimap v2.2.2. 5-letter seq is blue, WGBS is orange and EM-seq is green. The x-axis is the read depth and the curves plot the percentage of bases which achieve a certain read depth in each technology.

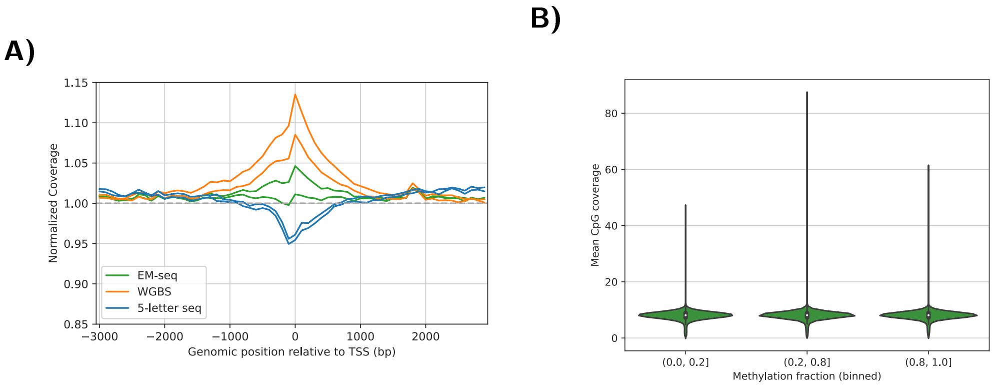

Supplementary Figure 4

A) Normalized coverage of CpGs around transcription start sites (TSSs). Normalized coverage is computed by dividing the coverage of each CpG by the mean coverage across all CpGs in the genome. Data is presented for all 6 samples (two replicates per technology) used in Figure 2; B) Violin plots represent the distribution of mean CpG coverage, pooled for the two 5-Letter seq datasets in 1kb windows, binned by the mean methylation level within each window. The distribution of coverages in each group is very similar with means and standard deviations, 7.98 ± 1.63, 8.0 ± 1.6, 7.99 ± 1.52, and medians (8.10, 8.11 and 8.1) respectively.

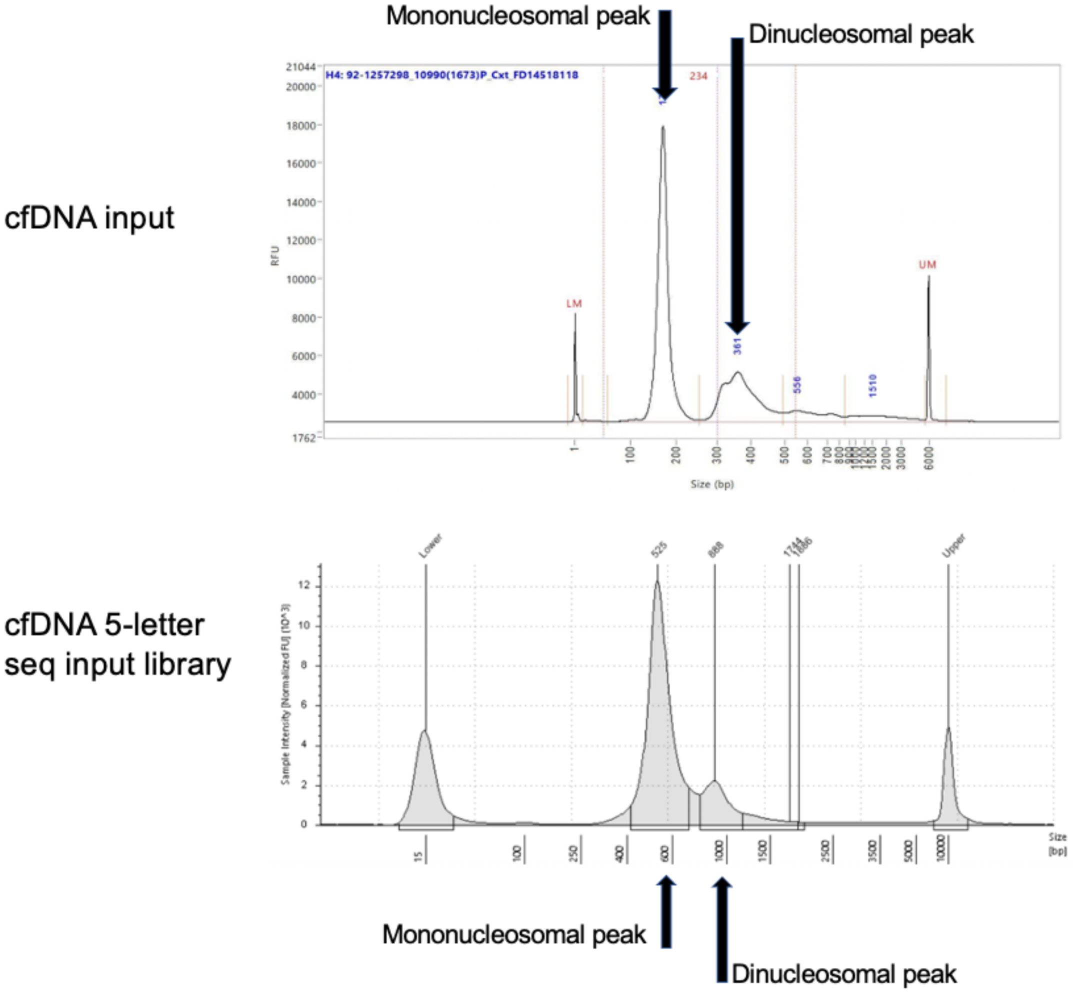

Supplementary Figure 5

Top: Fragment analyzer trace of cfDNA extracted from a double spun plasma sample from a single donor (Gastric cancer, stage IB) showing mono- and dinucleosomes.

Bottom: Tapestation trace (D5000) of the final 5-Letter seq library using 10ng of the above input. The mono- and dinucleosomal pattern of the input DNA is maintained in the final library. The final library size corresponds to the duplicated original strand, the hairpin and the Illumina P5/P7 adaptor.

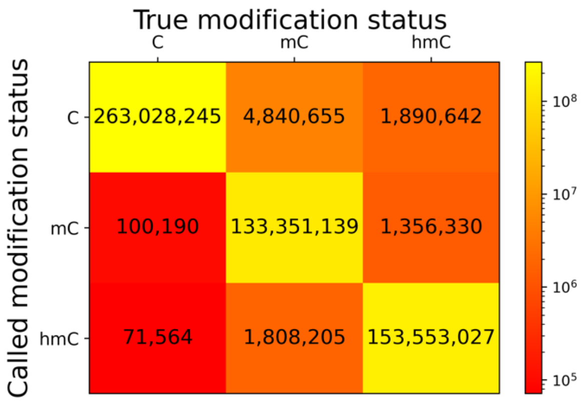

Supplementary Figure 6 The expected confusion matrix for base modification calls in a genome with mean CpG coverage of 10x and 5mC and 5hmC occurring a genome with 56 million CpGs with relative abundances of a 5hmC rich genome like that of neurons[1] where 5hmC is believed to occur at approximately 28%, 5mC at approximately 25%, and unmodC at approximately 47%. In this setting, we would expect to obtain highly accurate base modification calls with 98.8% of 5hmC calls occurring at sites which are indeed hydroxymethylated, 98.9% of 5mC calls occurring at sites which are indeed methylated, and 97.5% of unmodC calls occurring at sites which are indeed unmodified.

1. Wen, L. *et al.* Whole-genome analysis of 5-hydroxymethylcytosine and 5-methylcytosine at base resolution in the human brain. *Genome Biol.* **15**, (2014).

Supplementary figure 7

5’pGTATTGACAAGGTGACAGTATCGTCCAGGGACAGTCTCGTAGTACCACCTAGT
CTACTCGAGAATGTCAAGGTGTCAGAC 3’
3’
CATAACTGTTCCACTGTCATAGCAGGTCCCTGTCAGAGCATCATGGTGGATCAGA
TGAGCTCTTACAGTTCCACAGTCTGp 5’

C=5hmC
C=CpG

Supplementary Table 1

Metrics	5-letter seq	WGBS	EM-seq
PCR and cluster duplicate Rate	8.5%	2.68%	4.23%
Mapping Rate	89.77%	89.45%	92.03%
Mean coverage	15x	15.4x	16.7x
% covered at least once	90.21%	91.48%	91.05%
% covered at half mean coverage	87.82%	85.91%	87.48%
% resolved reads	98.42%	n/a	n/a
% errors in all reads	0.67%	n/a	n/a
% errors in resolved reads	0.45%	n/a	n/a

Supplementary Table 2

Metrics	2ng	10ng	80ng
PCR and cluster duplicate rate	38.2%	23.3%	14.4%
Mean coverage	21.8x	27.6x	30.4x
% covered at least once	90.9%	90.9%	90.4%
% covered at half mean coverage	87.78%	88.08%	87.98%
% resolved reads	98.07%	98.37%	98.37%
% errors in all reads	0.72%	0.70%	0.77%
% errors in resolved reads	0.48%	0.50%	0.56%
modC sensitivity (spike-in)	98.14%	98.18%	98.57%
modC specificity (spike-in)	99.90%	99.94%	99.98%

Supplementary Table 3

**Fragment length goal (bp)**	**Library yield (nM) - mean over 3 replicates**
176	27.6
222	25.17
245	23.77
319	27.17
400	20.17
